# Combating Antimicrobial Resistance With New-To-Nature Lanthipeptides Created by Genetic Code Expansion

**DOI:** 10.3389/fmicb.2020.590522

**Published:** 2020-11-05

**Authors:** Hamid Reza Karbalaei-Heidari, Nediljko Budisa

**Affiliations:** ^1^Department of Biology, Faculty of Sciences, Shiraz University, Shiraz, Iran; ^2^Department of Chemistry, University of Manitoba, Winnipeg, MB, Canada; ^3^Institute of Chemistry, Technical University of Berlin, Berlin, Germany

**Keywords:** genetic code expansion, chemoselectivity, RiPPs, lantibiotics, non-canonical amino acids, nisin, superbugs, synthetic biology

## Abstract

Due to the rapid emergence of multi-resistant bacterial strains in recent decades, the commercially available effective antibiotics are becoming increasingly limited. On the other hand, widespread antimicrobial peptides (AMPs) such as the lantibiotic nisin has been used worldwide for more than 40 years without the appearance of significant bacterial resistance. Lantibiotics are ribosomally synthesized antimicrobials generated by posttranslational modifications. Their biotechnological production is of particular interest to redesign natural scaffolds improving their pharmaceutical properties, which has great potential for therapeutic use in human medicine and other areas. However, conventional protein engineering methods are limited to 20 canonical amino acids prescribed by the genetic code. Therefore, the expansion of the genetic code as the most advanced approach in Synthetic Biology allows the addition of new amino acid building blocks (non-canonical amino acids, ncAAs) during protein translation. We now have solid proof-of-principle evidence that bioexpression with these novel building blocks enabled lantibiotics with chemical properties transcending those produced by natural evolution. The unique scaffolds with novel structural and functional properties are the result of this bioengineering. Here we will critically examine and evaluate the use of the expanded genetic code and its alternatives in lantibiotics research over the last 7 years. We anticipate that Synthetic Biology, using engineered lantibiotics and even more complex scaffolds will be a promising tool to address an urgent problem of antibiotic resistance, especially in a class of multi-drug resistant microbes known as superbugs.

## Antimicrobial Resistance and Superbugs

The use of antibiotics has enormously empowered modern medicine to save human lives and perform medical and surgical treatments under safe conditions (Aslam et al., 2018). In addition, treatment with antibiotics is one of the most important approaches to combat or prevent bacterial infections in animal populations and in the food industry. The widespread use of antibiotics began already during the Second World War, and Alexander Fleming himself was one of the first to recognize how improper use of these compounds could lead to antibiotic-resistant bacteria (Fleming, 1945). The habitats of our planet are saturated with various toxic substances, especially those of anthropogenic origin, which has contributed significantly to the selection of resistant strains (Davies and Davies, 2010). Indeed, the excessive use of antibiotics in agriculture, in the environment, animals (food, pets, aquatic fauna) and human medicine, and their release into the environment through fertilizer/feces is the most plausible cause of antimicrobial resistance (AMR) (Manyi-Loh et al., 2018).

The emergence of pathogens causing enhanced morbidity and mortality due to modifications in various physiological and biochemical mechanisms has led to multi-resistant microbes known also as “superbugs” (Coast et al., 1996). For example, in different microbial strains, resistance has emerged at multiple levels of defense, starting from the entry level (through the limitation of drug permeability and the existence of efflux pumps) to the degradation of the antibiotics via their modification or hydrolysis. Next, lateral gene transfer, alteration of binding through target modification and escape of toxicity through bypass reactions and metabolic shunts were (among others) documented as mechanisms for the development of single or multiple resistances (Yelin and Kishony, 2018). To sum up, due to the rapid appearance of multi-drug resistant bacterial strains in last few decades, the use of commercially available antibiotics has become increasingly limited. A promising alternative could be the use of antimicrobial peptides (AMPs) with many variants already in clinical trials (Table 1). Many bioactive peptides produced from various microorganisms (e.g., actinobacteria, cyanobacteria, bacilli, fungi) have a potential to serve as drugs against numerous pathogens (Greber and Dawgul, 2017; Ongey et al., 2017; Koo and Seo, 2019).

**TABLE 1:** List of selected promising AMPs in preclinical or clinical trials.

	AMP type	Mechanism	Application	Trial phase	References
Mutacin 1140	Lantibiotic	Lipid-II binding	Bacterial infection	Pre-clinical	Kers et al., 2018
NVB333	Lantibiotic	Lipid II binding	Bronchoalveolar infection therapy	Pre-clinical	Boakes et al., 2016
NVB302	Lantibiotic	Lipid-II binding	*Clostridioides difficile* infections	Phase I	Boakes and Dawson, 2014
Duramycin	Lantibiotic	Binding to Phosphatidylethanolamine	Cystic fibrosis, Inhibit cell entry of viruses	Phase II	Huo et al., 2017
DPK-060	Kininogen-derived peptide	Membrane targeting	Atopic dermatitis	Phase II	Håkansson et al., 2019
OP−145	LL−37 derivative	Bacterial membrane dissolution	Chronic leg ulcers, Wound healing effect	Phase II	Ming and Huang, 2017
D2A21	Synthetic	Unknown	Burn wound infections	Phase III	Chalekson et al., 2002
SGX942 (Dusquetide)	Synthetic	Innate defense regulator	Oral Mucositis	Phase III	Kudrimoti et al., 2017
PXL01	Lactoferrin analog	Repressing secretion of cytokines	Post-surgical adhesions	Phase III	Nilsson et al., 2009
Omiganan (CLS001)	Indolicidin derivative	Affect the cytoplasmic membranes of bacteria	Catheter-related infections	Phase III	Feng et al., 2015
POL 7080 (RG7929)	Protegrin analog	Inhibiting LptD	Septicemia, lung and thigh models	Phase III	Srinivas et al., 2010

## Antimicrobial Peptides

Antimicrobial peptides (AMPs) also known as host defence peptides are one of the most versatile natural products which represent alternatives to traditional antibiotics. In fact, AMPs are a special group of natural products that occur in almost every form of life, namely microorganisms, plants, and animals as an innate immune response (Ageitos et al., 2017). They can be used to treat various infections caused by pathogenic species with better efficacy, selectivity and specificity. Structurally, AMPs are versatile peptides with diverse lengths of almost always less than 80 amino acids in a linear or cyclic structure with a broad set of biotechnological applications, e.g., antibiotics, and inhibitors (Oliva et al., 2018).

**FIGURE 1:**
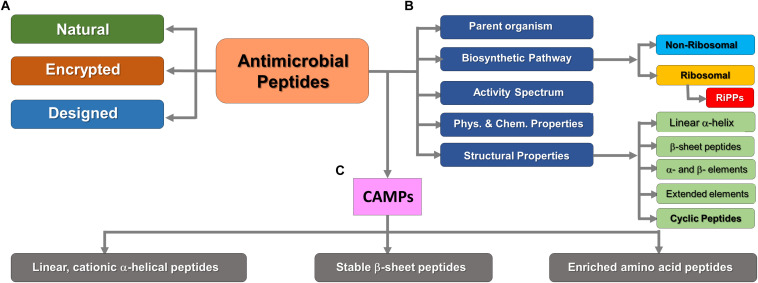
Classification of AMPs on the basis of various criteria. **(A)** Based on gene encoding pattern (Data-mining strategy). **(B)** Significant cellular or thermodynamic criteria. **(C)** Subdivision of positively charged (cationic) AMPs. RiPPs are structurally diverse but exclusively synthesized by ribosomes. This figure was compiled from a variety of sources (Porto et al., 2017; Koehbach and Craik, 2019).

Antimicrobial peptides can be classified on the basis of their parent organism, their biosynthetic production pathway (e.g., ribosomal or non-ribosomal origin), their activity spectrum, and their structural features (usually based on predominant secondary structure) or physicochemical properties (e.g., cationic/anionic, hydrophilic/hydrophobic etc.). They are structurally divided into five main classes, including (i) linear α-helical peptides, (ii) β–sheet-containing peptides, (iii) peptides with α- and β-structural elements, and (iv) extended or non-αβ secondary structure elements (e.g., polyproline helices) (Wang, 2015). Furthermore, they also occur in the form of (v) cyclic peptides and other topologically complex scaffolds, which are often ribosomally produced and post-translationally modified peptides (RiPPs) (Koehbach and Craik, 2019). Another classification system divides AMPs into natural, encrypted (Brand et al., 2012), and designed AMPs which are a product of specific genes or as part of larger proteins released after proteolysis, and the artificial ones developed by rational design techniques, respectively (Cardoso et al., 2016; Porto et al., 2017). Figure 1 summarizes the various methods of AMPs classification in the current literature.

**FIGURE 2:**
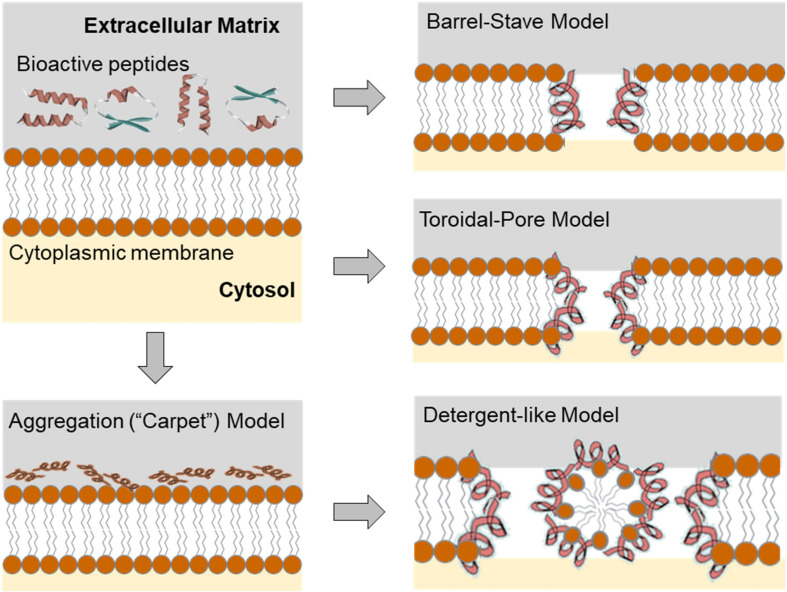
Configuration of the target membrane environment leading to the initial electrostatic and hydrophobic interactions of the AMPs on the bacterial membrane. Initial molecular event of the effect of the AMPs leads to the different scenarios that can be described using transmembrane pore and non-pore models. The carpet model, without pores: AMPs aggregate on the bilayer surface resulting in detergent-like disintegration, whereby the membrane is fragmented into micelles. Conversely, in the “barrel stave” model, membrane pores are formed by interactions between the hydrophobic surface of the pore and the acyl chains of the lipid core of the bilayer. The “toroidal pore” model (wormhole) combines the effects described by both the barrel stave and the carpet model. For a more comprehensive overview see Sun et al. (2018). However, it should be noted that the different mechanisms of AMPs action presented here do not cover the entire range of ribosomally produced and post-translationally modified peptides (RiPPs, *vide infra*), since not all members of this group damage membranes or form pores, but have different targets and mechanisms of action.

Three models (Carpet/Aggregation/Detergent-like model, Toroidal model, and Barrel-Stave model) have been proposed to explain the mechanism of action of AMPs for the ability to kill bacterial pathogens via membrane permeability (Figure 2). In all these models AMPs act in two sequential steps (Brogden, 2005). In the first step, electrostatic interactions are established between the positively charged amino acids in CAMPs with the negatively charged membrane surface, such as the pyrophosphate group of lipid-II in the cell membrane of microorganism or phosphatidylethanolamines in the outer leaflet of the target cell membrane. In the second step, the formation of pores within the membrane causes the bilayer to break open, which leads to extensive leakages and finally to cell death, particularly in Gram-positive bacteria. Detailed descriptions of detergent-like effects of amphipathic cationic AMPs and other models of membrane disruption by AMPs are described in great detail in the contemporary literature (see e.g., Zweytick et al., 2006). Achievable chemical diversity and variability in the production of peptides together with almost unlimited sequence space for matching their physicochemical properties and generating broad-spectrum antimicrobial activity make them a valid alternative to antibiotics in the effort to combat multi-drug resistant strains and save human lives in the face of future epidemic and other challenges (Ageitos et al., 2017). Nevertheless, several obstacles remain to be overcome in order to develop AMPs for medical use, such as toxicity, stability, and even bacterial resistance. Another obstacle is the lack of standard experimental procedures for quantifying AMP activity and we still do not have a clear picture of several AMP’s mechanisms of action (Torres et al., 2019).

## Lanthipeptides Are Ribosomally Produced and Post-translationally Modified Peptides (RIPPS)

RiPPs are an important class of AMPs, ribosomally synthesized and post-translationally modified bio-active peptides in all three domains of life and show great structural diversity. They are subdivided into more than twenty well-known categories including lanthipeptides, linaridins, proteusins, linear azol(in)e-containing peptides (LAPs), cyanobactins, thiopeptides, bottromycins, microcins, lasso peptides, microviridins, sactipeptides, bacterial head-to-tail cyclized peptides, amatoxins and phallotoxins, cyclotides, orbitides, conopeptides, glycocins, autoinducing peptides (AIPs), pyrroloquinoline quinone (PQQ), pantocin A and thyroid hormones, etc. (Arnison et al., 2013).

In this context, lanthipeptides are classified as one the largest group of RiPPs assigned in class I of bacterial-origin AMPs (bacteriocins). They are transformed into a mature active form by special post-translational modifications (PTMs) carried out by a battery of dedicated enzymes. Class I of bacteriocins include lantibiotics, linear azol(in)e-containing peptides (LAPs), thiopeptides, bottromycins, glycocins, lasso peptides, head-to-tail cyclized bacteriocins, sactibiotics, and lipolanthines (Acedo et al., 2018). Bacterial lanthipeptides exhibit outstanding properties such as conformational rigidity, increased metabolic and chemical stability as well as intense cell permeability that distinguish them from other heat-stable (class-II) and thermo-labile (class-III) bacteriocins (Alvarez-Sieiro et al., 2016). In addition to the four earlier types of lanthipeptides classified based on the biosynthetic mechanisms involved in their structural maturation, the recently discovered modified peptides, including Cocaoidin and Lexapeptide, exhibited novel modification features such as different biosynthetic gene clusters and have been introduced as class V lanthipeptides (Ortiz-López et al., 2020; Xu et al., 2020).

In general, lantibiotics show an efficient ability to kill Gram-positive bacteria and are particularly important for those that exhibit drug resistance such as methicillin-resistant *Staphylococcus aureus* (MRSA), *Streptococcus pneumoniae* (MRSP), vancomycin intermediate *S. aureus* (VISA), vancomycin-resistant *enterococci* (VRE), *Clostridioides difficile*, etc. Instability and/or insolubility at physiological pH, susceptibility to proteolytic degradation, and a low level of their production are features that mainly limit the use of lantibiotics in the clinic. It should also be noted that the antimicrobial activity of lanthipeptides is well understood and characterized in some RiPPs such as nisin (achieved by lipid-II binding) (Kuipers et al., 1996). Later, the lipid-II binding activity of some lantibiotics was also demonstrated (Wiedemann et al., 2001; Breukink and de Kruijff, 2006), although in some of them the mechanisms of action have yet to be clarified. Nonetheless, low cytotoxicity of lantibiotics to human cells due to the absence of their main target lipid II or negative net charge in eukaryotic membranes (Lagedroste et al., 2019) along with a few examples of naturally occurring resistance (Draper et al., 2015; Khosa et al., 2016a,b; Clemens et al., 2018) make lantibiotics excellent lead compounds for development of new therapeutic options.

## The Feasibility of Genetic Engineering to Produce More Potent Lantibiotics

### RiPPs Recombinant Expression for Classical Engineering—Nisin as an Example

A wide range of technologies are at our disposal for producing a peptide such as chemical synthesis, recombinant DNA technologies, and *in vitro* translation systems. The choice of a strategy for gene-encoded peptide production is largely determined by their size and chemical complexity. Peptides with sophisticated PTMs-generated structures such as RiPPs are preferably produced biosynthetically using host expression cells. Host cells with installed and functional PTM enzymes should be able to express RiPPs in an intact active form with distinctive architecture dominated by lanthionine bridges. In this context, the absence of a widely used large-scale production platform for lantibiotics with consistent quality is one of the main drawbacks of their therapeutic application.

There are generally two options for recombinant expression of lantibiotics, homologous or heterologous expression systems. Homologous expression strategies have limited use in particular due to the difficulty of cultivating the original producers (Maffioli et al., 2015; Mohr et al., 2015) and/or the difficulty of lantibiotics expression induction under laboratory conditions (Wescombe et al., 2011; Garg et al., 2012). Conversely, heterologous biosynthesis with host cells such as *Lactococcus lactis*, *Bacillus subtilis*, *Bacillus cereus*, and *E. coli* are normally preferred and, in many cases, well established. So far, several genetically manipulatable *Streptomyces* strains, including *Streptomyces lividans* (Ahmed et al., 2020), *S. albus* (Myronovskyi et al., 2018), *S. coelicolor* M145 (Gomez-Escribano and Bibb, 2011), *S. venezuelae* ATCC 15439 (Kim et al., 2015), *S. avermitilis* (Komatsu et al., 2010) have been reported to synthesize natural products from genetically engineered biosynthetic gene clusters. However, *Escherichia coli* is still the most common and attractive option. A list of successful examples of recombinant expression of different RiPPs families in heterologous hosts such as *E. coli* and *Streptomyces* strains were recently compiled by Zhang and colleagues (Zhang et al., 2018). Heterologous production of lantibiotics is generally conducted as inactive form of the antimicrobial peptide (pre-lantibiotic) to prevent any detrimental effects on host cell growth and viability.

In addition, there is great interest to express lantibiotics in microbial host that should facilitate a straightforward and efficient engineering (Kuipers et al., 1996). Numerous conventional protein engineering approaches such as site-directed mutagenesis, directed evolution and various computational tools aid synthetic biologists and biochemists to selectively diversify the properties of expressed therapeutic peptides. These include manipulations of thermodynamic stability, increased bioavailability, reduced aggregation or enhanced specificity and proteolytic stability (Adhikari et al., 2019). In addition, the implementation of *in vitro* and *in vivo* approaches in combination with genome mining data and high-throughput screening strategies has opened up unprecedented opportunities to modify and even improve antimicrobial activity, manipulate the physicochemical properties and widening of the antibacterial spectrum in the production of lantibiotics (Field et al., 2015). Recently an attempt to design and biosynthesize a two-lipid II binding motifs-containing lantibiotic, called TL19, is the latest example showing 64-fold stronger activity against *Enterococcus faecium* than nisin (Zhao et al., 2020).

The potential of classical protein engineering in complex RiPPs is perhaps best illustrated by recombinant nisin, well known for its broad-spectrum antibacterial activity produced in certain strains of *L. lactis* (Lubelski et al., 2008). Nisin as the most widely used lantibiotic in the food industry over the past 50 years has undergone various methods of bioengineering with an aim to improving its function and/or physicochemical features (Shin et al., 2016). To expand the scope of its activity, several bioengineered variants of nisin have been generated by site-directed mutagenesis and by classical chemical modifications (Ross et al., 2012), including *in vitro* chemical synthesis (Knerr and van der Donk, 2012; Koopmans et al., 2015). For example, the use of the saturation mutagenesis approach has led to the production of nisin S29 derivatives with increased activity against a number of Gram-positive antibiotic-resistant pathogens. In addition, the increased antimicrobial activity was generated in comparison to the wild type nisin A when tested against Gram-negative food-borne pathogens (Field et al., 2012). Moreover, recombinant production of the nisin Z mutants N20K, M21K, N27K, H31K improved peptide solubility at alkaline pH (Rollema et al., 1995). Next, residue alterations at distinct locations enabled the improvement of antimicrobial activity (Islam et al., 2009; Healy et al., 2013; Geng and Smith, 2018), the enhancement of diffusion through complex polymers (Rouse et al., 2012), and widening effect on some Gram-negative bacteria (Field et al., 2012).

**FIGURE 3:**
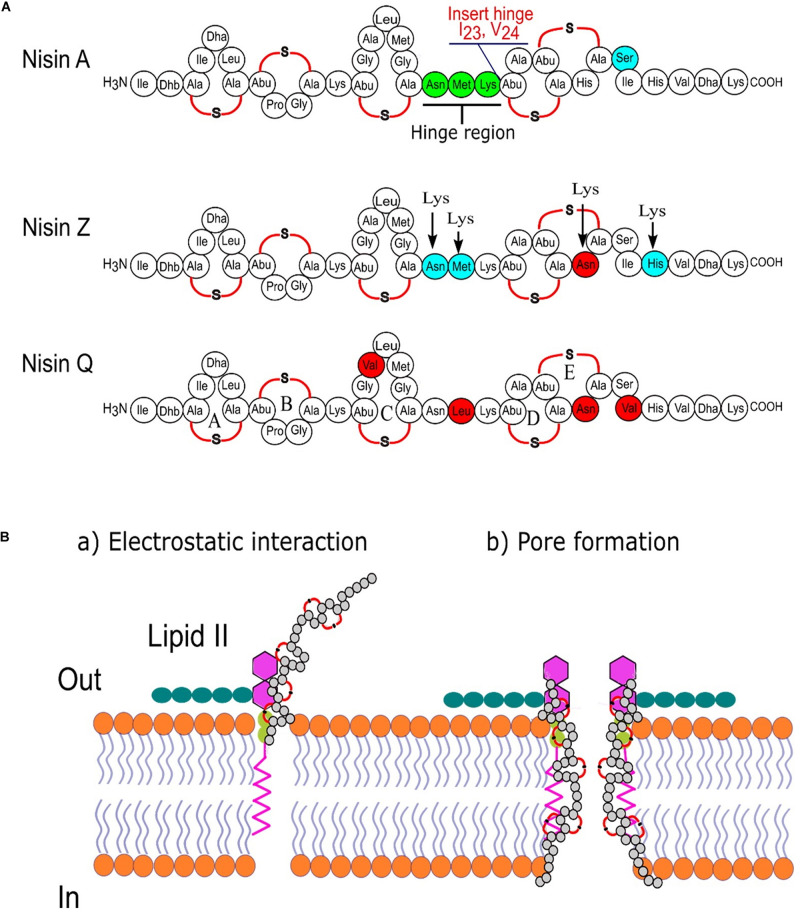
Nisin is one of the best-studied ribosomally synthesized, pore-forming, cationic, antimicrobial peptides. **(A)** Sequence comparison of three nisin variants from *L. lactis* and marking the important regions of nisin targeted for bioengineering by classical molecular biology approaches (Chatterjee et al., 2005); **(B)** two-steps mode of action of nisin.

Studies on the effects of the hinge region (NMK) length between rings C and D on antimicrobial activity and host specificity of nisin was also performed (Zhou et al., 2015). Although most variants with shorter or larger hinge length are less active than the wild type, some variants (+2, +1, −1, −2) exhibited higher antimicrobial activity than the wild type nisin A in agar-well-diffusion assays against *L. lactis* MG1363, *Listeria monocytogenes*, *Enterococcus faecalis* VE14089, *Bacillus sporothermodurans* IC4, and *Bacillus cereus* 4153. In addition, an extended nisin A variant of the hinge region (_2__0_NMKIV_24_) has been introduced, bypassing the human pathogen’s lantibiotic resistance while showing a slight decrease in antimicrobial activity (Zaschke-Kriesche et al., 2019). In this context, Figure 3 represents different variants of nisin produced by classical protein engineering together with a graphical representation of the mechanism of action of this bioactive peptide.

However, the expression of lantibiotics in popular prokaryotic hosts like *E. coli* is challenging as these bacteria have no enzymes to perform suitable PTMs to convert pro-peptide into a mature bioactive peptide. Therefore, their recombinant expression has to be coupled with the co-expression of active PTM biosynthetic gene clusters either *in vitro* or *in vivo*. These strategies are not in the focus of our study and interested readers are directed to numerous studies and reviews dedicated to this topic (Zhang et al., 2018; Myronovskyi and Luzhetskyy, 2019). We are mainly concerned with the *in vivo* design strategies that focus to expand the functional scope of AMPs from ribosomal templates beyond the classical protein engineering approaches.

### Beyond Classical Protein Engineering: Expanding the Scope of Protein Translation

It was argued that chemical and functional diversity delivered by PTMs in AMPs such as lantibiotics can be further supplemented or even expanded by the co-translational insertion of non-canonical amino acids (ncAAs) (Budisa, 2013). Indeed, a limited and conservative set of 20 canonical amino acids used by ribosomes to encode polypeptides in nature usually does not cover enough chemical space required to substantially expand their functional and structural diversity. In nature, this is normally achieved by site-selective PTMs that create special non-canonical amino acid side chains such as dehydroalanine (Dha) and dehydrobutyrine (Dhb) in lantibiotics (Figure 3). To directly mimic these and similar PTMs, chemists used, e.g., rhodium-catalyzed arylations (Key and Miller, 2017), P450-catalyzed cyclopropanations (Gober et al., 2017), and photocatalytic activation (de Bruijn and Roelfes, 2018) in thiopeptides, as well as metalloporphyrin-catalyzed alkylation of methionine in nisin (Maaskant and Roelfes, 2019) as attempts to obtaining novel AMPs with improved properties and/or activities. However, it is difficult to mimic PTM machinery chemically, whereas classical peptide synthetic protocols such as solid-phase peptide synthesis (SPPS) could not cover the chemical complexity of these natural products.

**FIGURE 4:**
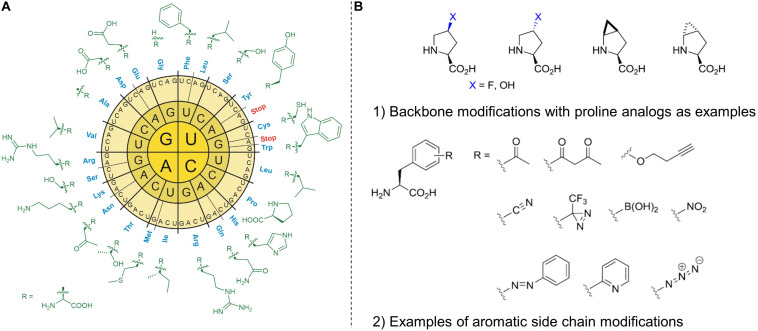
The radial arrangement of the standard genetic code in RNA format **(A)** with the side chains of 20 canonical amino acids (cAA). The standard amino acid repertoire of the genetic code can be modified or expanded **(B)** by non-canonical amino acids (ncAAs). By reprogramming protein translation, the ncAAs can be incorporated into recombinant proteins using *in vivo* and *in vitro* methods. These insertions offer useful modifications that can be made to the protein main chain (backbone) and the amino acid side chains (aliphatic or aromatic). Modified backbones and side chains often have unique physicochemical properties with the potential to dramatically expand the chemical and functional space of ribosomally synthesized peptides and proteins.

Therefore, the use of recombinant DNA technology that operates with heterologous expression of biosynthetic gene clusters and enriched with reprogrammed protein translation (Figure 4) can provide a reasonable solution to the above-mentioned problems (Hoesl and Budisa, 2012). It enables specific insertion of the biological, chemical, and physical properties delivered by ncAAs that can be accurately defined by the chemist at the bench. Cells equipped with various bioorthogonal chemistries have the potential to perform catalytic transformations that are not found in biology, including the creation of new metabolic and informational pathways (Devaraj, 2018). These modifications can also serve as redox sensors, spectroscopic markers (e.g., spin-labeling) or sensors of protein-ligand interactions. Metal-binding amino acids could lead to new structural, catalytic, or regulatory elements in proteins, while diazirines allow site-specific photo-crosslinking of the target protein to its substrate. Photoactive and photo-isomerizable ncAAs with novel photo-physical properties such as azobenzene side chains can be used to photo-regulate protein activity. Similarly, photocaged ncAAs can be activated by light to turn on enzymatic activity spatiotemporally (for review see Young and Schultz, 2010).

Directed evolution of aminoacyl-tRNA synthase (aaRS) plays a key role in the generation of such molecular tools. Desired ncAAs can be incorporated in a site-directed manner by reading in-frame stop codons, usually amber stop codons (UAG) with a suitable orthogonal pair (o-pair). Such a pair consists of an aaRS enzyme to activate the ncAA and its associated orthogonal tRNA (*vide infra*). However, the transition to recombinant production with such a possibility by using heterologous expression hosts is not trivial. For that reason, the residue-specific replacement of a particular amino acid at all positions in a protein sequence (known as selective pressure incorporation, *vide infra*) is a reasonable alternative as it does not require o-pairs (Budisa, 2004). Finally, the capacity of ncAAs incorporation should not interfere with the sequence of posttranslational modifications leading to the formation of lantibiotics and the restoration of their bioactivity.

With such systems in hands we would have very sophisticated tools to rationally engineer RiPPs scaffolds as now they can be redesigned by reprogrammed protein translation which utilize various ncAAs. This approach would have many advantages over the above discussed classical chemical or recombinant approaches as it enables: (i) genetic encoding of desired ncAAs and its sequence positioning; (ii) recombinant production under mild conditions at room temperature and atmospheric pressure; (iii) the targeted functionalization (e.g., various site-directed bioconjugations, light induced cross-linking, metal or cofactor binding, fluorophore or pharmacophore attachment, adhesiveness, etc.) (Cropp and Schultz, 2004). Crucial requirements to expand the scope of protein synthesis with ncAAs should be cellular uptake, their intracellular metabolic stability and translational activity (i.e., incorporation). Finally, it is necessary to reallocate (reassign) coding triplets (codons) in the genetic code to insert ncAAs in target protein/peptide sequences.

## Recombinant Lanthipeptides Produced by Incorporating Non-Canonical Amino Acids

### Reprogrammed Protein Translation for Chemoselective Labeling of Lantibiotics

**FIGURE 5:**
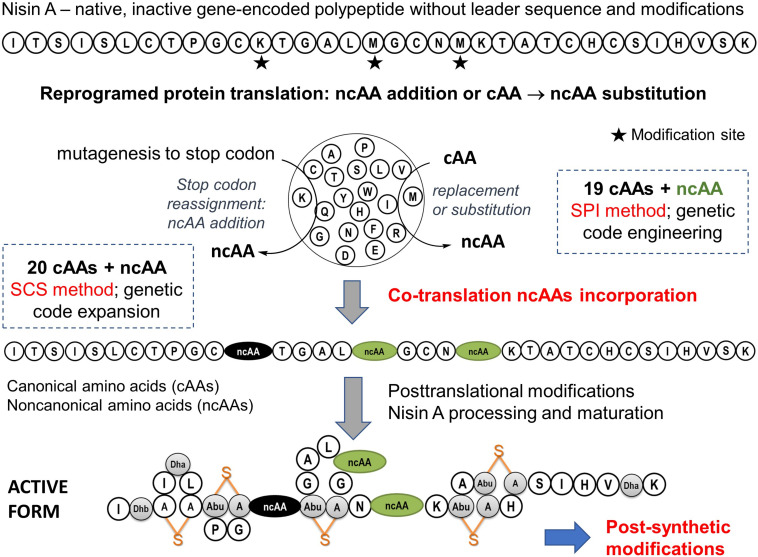
Expansion of scope of the nisin A biosynthesis by ncAAs. The biosynthetic production of active nisin involves the expression of peptide precursors with 20 canonical amino acids as standard building blocks, followed by posttranslational modifications (PTMs). The active form of natural nisin is characterized by the presence of unusual posttranslationally introduced bridge structures known as lanthionines (depicted in orange). It is generally expected that the incorporation of ncAAs (i.e., co-translational modifications) along with post-synthetic modification options (e.g., click chemistry) will create additional functional and structural levels of RiPPs diversification. For example, new variants with improved properties such as stability, specificity, bioavailability, action spectrum and half-life could be created. This is particularly important in view of the fact that nisin is highly effective against a number of human clinical pathogens, including many multi-drug resistant strains (Piper et al., 2009). In recent years it has been clearly demonstrated that several variants of nisin increase activity or stability in different experimental setups (reviewed in Field et al., 2015).

The bioexpression of lantibiotics is not fully comparable to the routine recombinant expression of soluble proteins because these polypeptides contain precursor (consisting of leader and core peptide) and recognition sequences. The core peptide is modified by post-translational modification (PTM) enzymes and upon proteolysis and export, transformed into a complex natural product. Unlike non-ribosomal natural products, native RiPPs cannot explore amino acids beyond the canonical 20 proteinogenic amino acids in the synthesis of precursor peptides, limiting their structural diversity at this level of ribosomal synthesis. On the other hand, from a biotechnological point of view, the great advantage of the ribosomal synthesis (coupled efficiently with PTMs) of bioactive compounds would be the possibility of achieving high chemical diversity at low genetic cost and to avoid supply of expensive precursors. The drawback that many unusual building blocks (found in non-ribosomal synthesis) are absent in RiPPs can be efficiently circumvented by genetic code expansion (GCE). The insertion of ncAAs (in different combinations of their numbers and chemistry) into growing peptide represents a novel level of chemical diversification of these sequences (Figure 5). Incorporated ncAAs can either directly interfere with PTMs or occupy sequence positions not affected by the PTM machineries. This provides suitable bio-orthogonal reactive groups (‘handles’) amenable for various chemoselective ligand couplings. In addition, both site- and residue-specific incorporation techniques generally allow for fine chemical manipulations of the amino acid side chains, e.g., of Pro, Trp, Tyr, Met and to visualize them in spectroscopic recordings (Baumann et al., 2018).

Non-canonical amino acids equipped with specific chemical groups (azides, olefins, ketones and aldehydes, loaded and unloaded alkynes, halogens, oximes, hydrazones, boronic acid esters and acids, etc.) give polypeptide sequences a unique reactivity and chemoselectivity that allow easy and efficient bioconjugations to a variety of ligands (Agostini et al., 2017). The copper(I)-catalyzed Huisgen cycloaddition reaction between azides and alkynes (also known as “click chemistry”) is widely used to functionalize labeled proteins since it takes place under physiological conditions allowing full retention of the protein structure (Kolb et al., 2001). For example, click chemistry proved to be efficient in producing nisin-peptoid hybrids for therapeutic use (Bolt et al., 2018). Further, *in vitro* metathesis reaction confirmed the capability of the variants for post-biosynthetic modifications such as conjugation reactions with ligands, labels, or tags using bio-orthogonal chemistry. Other alternatives include recently developed very efficient chemoselective methods; mainly copper-click, photoclick, and catalyzed oxime/hydrazone chemistries (Devaraj, 2018). This methodology could provide the RiPPs derivatives with an increase in chemical diversity such as enhanced proteolytic resistance, or increased bioavailability. Finally, bioexpression with various ncAAs combined with the possibilities for further chemical processing of RiPPs after maturation (i.e., semi-synthesis of mature products) makes the number of possible chemical combinations in antibiotic design virtually limitless.

**FIGURE 6:**
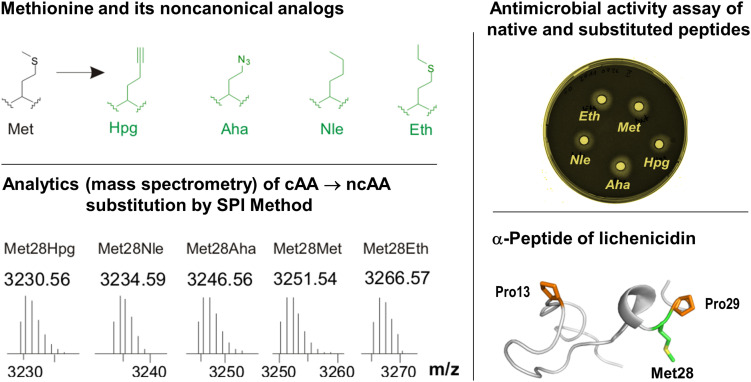
Incorporation of Met analogs into the one subunit of the two-component lantibiotic lichenicidin using the SPI method. Top left: Structures of methionine and its analogs. Met, L-methionine; Aha, L-azidohomoalanine; Hpg, L-homopropargylglycine; Nle, L-norleucine and Eth, L-ethionine. These analogs were used to individually replace Met residues in the precursor peptide of lichenicidin (bottom right), which was also analytically confirmed (bottom left). The substituted α-peptide of lichenicidin underwent PTM-activity, whereby the linear precursor peptide was converted into a polycyclic form that exhibits antimicrobial activity when mixed with the β-peptide (top right). For more details see Oldach et al. (2012). Most recently, Kuipers and co-workers have demonstrated the use of click chemistry in the engineering of nisin by using SPI with the methionine analogs Hpg and Aha (Deng et al., 2020).

To gain access to such a sophisticated chemistry in recombinant proteins, the incorporation of suitable ncAAs must be established in order to have possibilities for the site- or residue-specific functionalization of e.g., bioorthogonal alkyne- or azide-containing moieties. The simplest strategy for the *in vivo* insertion of ncAAs is based on the use of auxotrophic microbial systems and is known as selective pressure incorporation (SPI) (Minks et al., 2000). This methodology also exploits natural substrate tolerance (“substrate promiscuity”) of protein translation machinery components toward ncAAs analogs. For example, with a Met-auxotrophic *E. coli* strain in Met-depleted medium, it was possible to express proteins in which methionine was replaced by different medium-supplemented Met analogs with a terminal azide or alkyne as a side chain (Figure 6). The incorporation protocol typically requires the addition of the ncAA analog in the growing medium, which is taken up by the cell machinery and used in protein translation (Budisa, 2004). Furthermore, the SPI is well known for higher translation yields without the need for extensive host engineering.

The SPI method essentially requires a genetically and metabolically stable auxotrophic expression host with ncAA recognized and activated by endogenous translation apparatus. Efficient and robust protein expression systems with controlled fermentation and expression conditions usually yield target proteins often similar to the wt-counterparts. This was demonstrated in 2012, when the first application of the SPI method for RiPPs was reported (Oldach et al., 2012). In particular, the residue-specific incorporation of various Trp, Met, and Pro analogs into the modular two-component lantibiotic lichenicidin proved to be fully attainable (Figure 6). In this system, the plasmid-encoded SPI-based expression of modular propeptides is coupled with the expression of the fosmid-encoded PTM machinery in the *E. coli* host cells. It has also been shown that lichenicidin with the Met analog homopropragylglycine (Hpg) can be post-synthetically functionalized by click chemistry using azidofluorescein as a ligand.

**FIGURE 7:**
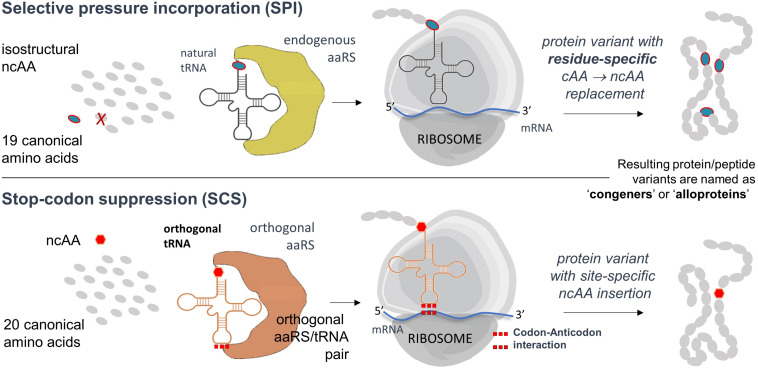
Flow chart of the SPI and SCS methods. Both methods can be performed separately or combined in a single expression experiment (Hoesl and Budisa, 2011). Further details, advantages and disadvantages of both methods are described in the text.

However, the fundamental disadvantage of the SPI method is that it allows residue-specific substitution of the canonical amino acid of interest by isosteric analogs, uncontrolled substitutions in the entire proteome, possibly endangering the heterologous PTM machinery and thus damaging the host expression cell. Furthermore, often in this approach the incorporation of ncAA is achieved in a statistical manner, which leads to a heterogeneity of the protein samples. This can be circumvented by using site-directed incorporation methods (Figure 7). However, if a site-directed ncAA incorporation is desired, the protein translation must be orthogonalized. A specific position in the protein to be labeled is mutated to the in-frame stop (usually amber) codon in the target gene. An essential part of this approach is therefore the introduction of in-frame stop codons (UAG) in target mRNAs decoded by the ribosome. In-frame stop codon suppression (i.e., readthrough) is achieved via a specially designed suppressor tRNA charged with ncAA (by a dedicated enzyme) and leads to the ncAA incorporation in a site-specific manner. This expands the amino acid repertoire of the genetic code and provides a basis for the technology known as stop-codon suppression (SCS) also as GCE. GCE generally requires the introduction of orthogonal aminoacyl-tRNA synthetase:tRNA pairs (o-pairs) into recombinant expression systems. Heterologous expression systems operating with orthogonal pairs are also known as orthogonal translation systems (OTS) in the literature. This research area is very well covered by a number of recent reviews (see, for example, Soye et al., 2015; Arranz-Gibert et al., 2018).

In orthogonal translation, usually a TAG (amber) stop codon is assigned to the ncAA and the orthogonal tRNA anticodon is mutated to CUA for position-specific incorporation. Orthogonal pairs of archaeal origin can be into eubacterial (such as *E. coli*) or eukaryotic hosts (such as *Saccharomyces cerevisiae*) and used to affect orthogonal translation (Wang et al., 2006). In particular, to engineer o-pairs, it is necessary to redesign an existing aaRS to activate the desired new ncAA substrate. Commonly used orthogonal pairs are based on *Methanocaldococcus jannaschii* tyrosyl-tRNA synthetase (*Mj*TyrRS) and *Methanosarcina mazei/barkeri* pyrrolysyl-tRNA synthetases (*Mm*PylRS or *Mb*PylRS). Compared to *E. coli* or eukaryotic hosts they show a distant phylogeny (due to their archaeal origin) that allows their implementation as o-pairs (i.e., they do not show cross-reactivity with the endogenous translational apparatus) (Budisa, 2006). Enzyme variants are generated by mutagenesis of the plasmid library and selected iterative positive/negative selection cycles (Bryson et al., 2017). This technology enables the incorporation of ncAAs containing different chemical entities into protein, both at one position (site-directed) and at several positions (multi-site mode). Recently, we have developed a computer-aided approach (Baumann et al., 2019) to create highly diversified libraries of aminoacyl-tRNA synthetases (AARSs) and have demonstrated the engineering of orthogonal translations with up to 50-fold improved insertion efficiency. This was made possible by creating much larger libraries (with > 10^9^ clones). In conventional directed evolution protocols, only about 10^7^ clones are generated.

We and others have already applied and optimized orthogonal pairs for site-specific incorporation of fluorinated amino acids (Völler et al., 2015). Recently, we also have developed a novel and highly efficient orthogonal tyrosyl-tRNA synthetase from *Methanocaldococcus jannaschii* (*Mj*TyrRS) for photo-activatable L-DOPA derivatives (Hauf et al., 2017). Similarly, we evolved a novel pyrrolysyl-tRNA synthetase from *Methanosarcina mazei* (*Mm*PylRS), which is able to incorporate S-allylcysteine (Sac) in response to amber stop codons (Exner et al., 2017). This *Mm-* or *Methanosarcina barkeri* PylRS (*Mb*PylRS) variants should be an ideal starting enzyme for the evolution of enzyme mutants with high specificity for smaller substrates. These and other advances in the field are recently summarized in a series of specific reviews (Arranz-Gibert et al., 2019; Budisa and Schneider, 2019) and highlights (Mayer, 2019; Yanagisawa et al., 2019).

Without a doubt, GCE will soon become a groundbreaking technology that will allow the catalysis of reactions that are not possible with canonical amino acids alone. More than 200 distinct ncAAs have been incorporated into various peptides and proteins so far (Vargas-Rodriguez et al., 2018). This technology has already yielded new-to-nature RiPPs and proteins in general with improved or novel physicochemical and biological properties which enabled the study of the structure-function relationship of proteins, imaging (Uttamapinant et al., 2015), probing (Chatterjee et al., 2013) and even the development of new therapeutics. Furthermore, it is possible to mask side chain functionalities of amino acids of interest by replacing them site-specifically with e.g., photo-caged ncAA. These masked substrates can then be non-invasively unmasked by UV light irradiation revealing critical functionalities (e.g., photo-caged residues which are substrates for *Mj*TyrRS and *Mm* or *Mb*PylRS-based orthogonal translation) (Drienovská and Roelfes, 2020). For the incorporation of ncAAs into RiPPs, various methods have been considered so far, ranging from *in vitro* synthesis reactions to residue and site-specific *in vivo* incorporation (Bartholomae et al., 2018). To maximize the yield and cost-effective production of lantibiotics, the biological synthesis of these compounds offers a viable alternative strategy with less downstream processing.

### GCE as a Technology for New-To-Nature Lanthipeptides: Troubleshooting and Some Insider Tips

The deeper understanding of the molecular mechanisms behind the activities of AMPs, their selectivity and rational manipulation requires the application of novel technologies of peptide modifications. Incorporation of ncAAs is a particularly promising tool, which is able to deliver new chemical functions and specific probes into peptide structures. For example, Nagao et al. reported the first example of heterologous expression of the type II lantibiotic, nukacin ISK-1 in *E. coli* by co-expression of NukA and the modification enzyme NukM (Nagao et al., 2005). In the following years, this approach was increasingly replaced by the insertion of different ncAAs that mimic different PTMs, such as the photocrosslinker *p*-Benzoyl-L-Phe (pBpa) in protein and proteome chemistry due to the orthogonal translation rapid development using different vectors and expression systems in *E. coli* (Miyazaki et al., 2018). In general, the ncAA-insertion provides many advantageous properties when compared with PTMs, such as functional diversity, probing and controllable activity. In this context, Table 2 represents a list of the new-to-nature RiPPs biosynthetically produced via GCE methodology.

**TABLE 2:** List of the new-to-nature RiPPs produced by genetic code expansion technology reported so far.

	Type	Source/Host	New RiPPs	Activity*or Peptide yield (mg/L)	References
Prochlorisin A 3.2	Class II lantibiotic	*Prochlorococcus*/ *E. coli*	F26-pBpa	Equal	Shi et al., 2011
Capistruin	Lassopeptide	Actinobacteria/ *E. coli*	I4- Alk	0.110	Al Toma et al., 2015
			I4-Nbk	0.039	
			I4-BocK	0.110	
			I4-Ack	0.042	
			I4-Pck	0.041	
			A10- Alk	0.290	
			A10-Nbk	0.218	
			A10-BocK	0.435	
			A10-Ack	0.149	
			A10-Pck	0.143	
			G17-Alk	0.033	
			G17-Nbk	< LoQ	
			G17-BocK	0.013	
			G17-Ack	< LoQ	
			G17-Pck	< LoQ	
MccJ25	Lassopeptide	*E. coli/E. coli*	V6, I13- *m*Br-F	Less	Piscotta et al., 2015
			F10, F19- *m*Br-F	Equal	
			V6, I13- *m*NO_2_-F	Less	
			F10, F19- *m*NO_2_-F	Less	
			V6, I13- *m*Cl-F	Less	
			F10, F19- *m*Cl-F	Equal	
			V6, I13- *m*CF_3_-F	Less	
			F10, F19- *m*CF_3_-F	Less	
Thiocilin	Thiopeptide	*B. cereus/B. cereus*	T3, T4, V6, T8, T13-BocK	Decreased	Luo et al., 2016
			T3, T4, V6, T8, T13- AlocK	Decreased	
			T3, T4, V6, T8, T13- ProcK	Decreased	
Nisin A	Class I lantibiotic	*L. lactis*/*E. coli*	S3 -Fluoro-pAcF	2.00	Zambaldo et al., 2017
			T8- Fluoro-pAcF	1.50	
			T13- Fluoro-pAcF	3.00	
Cinnamycin	Class II Type B lantibiotic	*S. albus*/*S. albus*	R2-Alk	Decreased	Lopatniuk et al., 2017
			F10-Alk	Decreased	
Nisin A	Class I lantibiotic	*L. lactis*/*L. lactis* *L. lactis*/*E. coli*	I4-BocK	Equal	Bartholomae et al., 2018
			K12-BocK	Equal	
			I4-BocK		
			K12-BocK		
Lacticin 481	Class II lantibiotic	*L. lactis/E. coli*	W19-*o*-Cl-F, W19-*m*-Br-F, W19-*o*-NO_2_-F, F21-*o*NO_2_-F, F23-*o*-Cl-F	Increased	Kakkar et al., 2018
			W19, F21, F23- *o*Br-F W19, F21, F23- *o*NO_2_-F W19, F21, F23- *m*Br-F W19, F21, F23- *m*CF_3_-F	Decreased	

ND, not determined. * Antimicrobial activity in comparison to wild-type; < LoQ = below the limit of quantification. L. lactis, Lactococcus lactis; S. albus, Streptomyces albus; pBpa, p-benzoyl-L-phenylalanine; Nbk, Nε-5-norbornene-2-yloxycarbonyl-L-lysine; Alk, Nε-Allocl-Lysine; Ack, Nε-2-azidoethyloxycarbonyl-L-lysine; Pck, Nε-2-propyn-1-yloxycarbonyl-L-lysine; Bock, Nε-Boc-L-lysine; mBr-F, meta-Bromo-phenylalanine; mNO2-F, meta-Nitro-phenylalanine; mCl-F, meta-Chloro-phenylalanine; mCF_3_-F, meta-trifluoromethyl-phenylalanine; AlocK, Ne-allyloxycarbonyl-L-lysine; ProcK, Ne-prop-2-ynyloxycarbonyl-L-lysine; pAcF, p-acetylphenylalanine.

We define lanthipeptides expressed recombinantly with ncAAs as “new-to-nature” because they are endowed with chemical functionalities that are normally a domain of classical organic chemistry. Most of such genetically encoded modifications introduced in proteins and peptides (*in vivo* and *in vitro*) take place on amino acid side chains and very seldom on protein backbones (like e.g., modified proline analogs; see Figure 4; Larregola et al., 2012). For example, D-amino acids, β-, or γ-amino acids, dipeptides and dipeptidomimetic analogs can be inserted into target polypeptide sequences by using engineered *in vitro* translation systems (Lee et al., 2019). Translation with backbone modification is difficult because ribosomes are being evolved to facilitate the polymerization of α-L-amino acids into polypeptides. Since the majority of the canonical amino acids can be considered to be derivatives of alanine (Kubyshkin and Budisa, 2019), the ncAA insertions (via SCS) or substitutions (via SPI) are mainly additions of new side-chain functionalities- a sort of useful co-translational addition to existing PTMs that should greatly expand the functional performance of modified peptides and proteins.

The engineering of orthogonal aminoacyl-tRNA synthetase/tRNA orthogonal pairs (o-pairs) for the site-specific incorporation of ncAAs is undoubtedly an important tool in protein and peptide design. However, it should be noted that orthogonalization of protein translation is usually associated with a significant decrease in system performance. Therefore, bio-expressions with orthogonal pairs require that the desired ncAA is available in large excess (Völler and Budisa, 2017). In addition, o-pairs based on both *Mj*TyrRS and *Mm*(*Mb*)PylRS scaffolds can have advantages and disadvantages in different environments. In bacterial host cells, *Mj*TyrRS often show a higher performance compared with PylRS-based systems (Rauch et al., 2016). In general, orthogonal translation with PylRS-derived o-pairs leads to lower yields of target protein, since this enzyme generally has a low catalytic efficiency (Baumann et al., 2016). While o-pairs based on *Mj*TyrRS allow a higher number of in-frame stop codons that can be suppressed, this is not the case with orthogonal translation based on PylRS (Zhao et al., 2018). Many strategies have been reported to alleviate these limitations, including the computational redesign of both enzymes as discussed elsewhere (Baumann et al., 2019).

The incorporation of ncAAs via SCS into proteins in general and RiPPs in particular is usually associated with reduced expression efficiency since the readthrough of stop codons is context-dependent. In particular, the insertion of in-frame stop codons in target sequences might generate so-called “context effects” with the competition with ribosomal release factors (RFs) for the readthrough (Plotkin and Kudla, 2011). Next, the insertion of a stop codon into a specific sequence is often associated with a high level of interference with natural mRNA folding (Gorochowski et al., 2015). This requires the optimization of mRNA sequences by applying a computer-aided design that should restore or even improve the strength of binding site interaction within the ribosome (Del Campo et al., 2015). For these reasons, “context effects” during the incorporation of ncAA in target sequences were investigated by randomly generating in-frame stop codons along the gene sequence with different proteins (summarized in Agostini et al., 2017).

The screening of an amber stop codon library with lantibiotic genes to study site-specific ncAAs incorporation efficiency could be of great importance to improve and enhance the biosynthesis of RiPPs with ncAAs. For example, we and others recently established a reporter platform for SCS approach using three expression vectors capable of independent control of different groups of genes. Each sense codon in the core peptide region of NisA was replaced by TAG (amber) codon and screening of possibly suitable incorporation positions was assessed upon incorporation of various analogs (Bartholomae et al., 2018). Next, in addition to the feasibility of a position to incorporate ncAAs, the admissibility of target genes that can fold and modify is critical and must always be considered. This is best illustrated by the report of Shi et al. (2011), in which the orthogonal translation was performed with three different types of bioactive peptides, prochlorosins from class II lanthipeptides, two-component lantibiotic haloduracin and the class I lantibiotic, nisin.

As discussed above, the residue-specific replacement of a particular amino acid at all positions in a protein sequence with ncAA (i.e., SPI method) proved to be a reasonable alternative in some cases (Hoesl and Budisa, 2011). Moreover, incorporation of three different tryptophan analogs (5-fluoroTrp, 5-hydroxyTrp, and 5-methylTrp) into different positions of nisin (I1W, I4W, M17W, and V32W), which included the overexpression of tryptophanyl-tRNA synthetase (TrpRS) in *L. lactis* strain PA1002, showed a reasonable incorporation efficiency and suitable production yield (Zhou et al., 2016). However, in a comparative study between the efficiency of ncAA incorporation in orthogonal translation and by SPI method into the lasso peptide capistruin, the SCS approach showed higher efficiency (Al Toma et al., 2015).

### Practical Expression, Purification, Monitoring Strategies, and Challenges With Novel Lanthipeptides

After induction and expression of the target gene, various conventional methods such as immobilized metal affinity chromatography (IMAC) and in rare cases, additional column chromatography were applied for the purification purposes. Due to the lack of a cell wall-anchored protease (NisP), and to avoid the requirement for immunity as well as to achieve higher production yield in *E. coli* (Montalbán-López et al., 2018), activation of the purified pre-lantibiotics is usually done by using native proteases or some commercial enzymes *in vitro*. To assess proper maturation of the lantibiotics, MS-analyses and bioactivity assays have been employed. In addition, RP-HPLC is one of the most convenient methods used for the measurement of the concentration before and after the activation by proteolytic cleavage of the leader peptide. Monitoring and quantification of the active lantibiotic by integrating the leader peptide peaks in RP-HPLC might be complicated due to alteration of running properties of the pre-lantibiotics and activated forms during the experiment and make it difficult to distinguish between similar peaks. This strategy has been used to determine the concentration of the activated lantibiotic during the production of genome-mining new lantibiotics as a hybrid containing the leader peptide of nisin (Lagedroste et al., 2019).

Another important issue is incomplete modification of target peptides such as lack of lanthionine ring formation or the defected dehydrations of Ser/Thr residues which lead to the disrupted recognition by the leader peptidases and severe decline of their antimicrobial activities. Although MS analysis of the cleavage products of pre-lantibiotics provides useful information about the dehydration reaction by the decrease of a water molecule mass (−18 Da), the technique cannot indicate the formation of methyl-lanthionine rings. In this regard, use of alkylation agents such as CDAP to bind the free cysteine thiol or peptide digestion approach using tandem MS-MS analysis to determine different patterns between wild type and mutant variants has recently been introduced (Kluskens et al., 2005). Verification of antimicrobial activity of recombinant lantibiotics can also be conducted by growth inhibition assay. Using lantibiotic-sensitive strains such as *L. lactis* strain NZ9000, *Listeria monocytogenes*, *Clostridioides difficile*, etc. have been reported in the literature (Field et al., 2010; Cebrián et al., 2019).

Several techniques have been developed for verification and quantification of nisin, such as turbidity assays (Berridge and Barrett, 1952), colorimetric assays (Wang et al., 2007) and agar diffusion bioassay (Mocquot and Lefebvre, 1956). Agar-based bioassay, as the most widely used method for quantifying nisin activity was implemented based on diffusion through agar gel seeded with nisin-sensitive indicator bacteria (Pongtharangkul and Demirci, 2004). The diameter of the inhibition zone generated by growth inhibition of indicator bacteria is correlated with the concentration of nisin. Factors such as nisin structure, concentration of agar, pH, existence of detergent, number of indicator cells and temperature can affect diffusion of nisin through agar. Several researchers have attempted to improve the accuracy, sensitivity, and reproducibility of conventional agar diffusion bioassays for nisin by optimizing several factors such as concentration of agar and buffering conditions, addition of a detergent like Tween 20, incubation time adjustment, and the temperature of pre-diffusion of the agar plates and even the size of wells on agar (Rogers and Montville, 1991; Wolf and Gibbons, 1996; Bonev et al., 2008; Lalpuria et al., 2013).

A modified form of agar diffusion bioassay was established upon activation of nisin by the membrane-associated protease enzyme (NisP). Antimicrobial activity of precursor lantibiotics and ncAA-modified variants synthesized by native producers or heterologously expressed by host cells could be determined using a sensitive indicator strain harboring appropriate genes for the maturation of the lantibiotic. For example, the nisin-sensitive indicator *L. lactis* NZ9000 pNZnisPT pIL253 showed reasonable applicability to assess functional and physicochemical properties of modified nisin variants like proline, tryptophan, and lysine incorporated analogs (Bartholomae et al., 2018). In addition, a bioactivity assay for two-component lantibiotics such as lichenicidin which requires the synergistic activity of two peptides was introduced which is called “deferred antagonist bioassay.” Using agar well diffusion plates by mixing the cell-free supernatants of both separately producing strains in suitable proportions against growth of the indicator strain and the measurement of inhibition radii after appropriate incubation at reasonable temperature aid us to easily screen a large library of lantibiotic mutants by avoiding time-consuming purification steps (Barbosa et al., 2019).

### Most Recent Advances in Recombinant Lanthipeptides Production With an Expanded Genetic Code

Bindman et al. (2015) have demonstrated that co-translational backbone modification α-hydroxy acidic insertion works in RiPPs in response to in-frame amber codons (SCS method). They have shown the possibility of lantibiotic activation after co-translational incorporation of α-hydroxy acids into the precursor peptides in *E. coli*. The biosynthesis of lacticin 481 or nukacin ISK-1 analogs by incorporating Boc-HO-1, HO-Phe(3-Br)-OH or HO-Tyr(propargyl)-OH at the junction of the leader peptide to the core region of the lantibiotics (position + 1) was successful. This enabled an improved removal step of the leader peptide in a general manner without the need to screen for various proteases (Bindman et al., 2015). Efforts have also been reported to increase the chemical diversity of lasso peptides and even to determine the crystal structure of these complicated cyclic peptides in the presence of bromine atoms (Piscotta et al., 2015). Sixteen possible variants were expressed in *E. coli* at four positions (Val6, Ile3, Phe10, Phe19). Thereby, four meta-substituted Phe derivatives (*m*-ClPhe, *m*-BrPhe, *m*-NO_2_Phe and *m*-CF_3_Phe) were incorporated into MccJ25 using an engineered “polyspecific” *Mm*PylRS. The results showed that the yield of ncAA-substituted MccJ25 variants intensively depended on the position and the chemical nature of the substitutions.

The use of ncAAs with 1,3- or 1,2-aminothiol reactive groups to promote the cyclization of a downstream target peptide sequence via a C-terminal ligation/ring contraction mechanism has also been reported (Frost et al., 2015). It has been demonstrated to be useful for the formation of macrocyclic side-chain-to-tail peptides *in vitro* in a pH-controlled manner. This strategy controls the spontaneous cyclization of peptides of variable length and completely random sequences with a wide range of molecular arrangements, namely cyclic, lariat or C-terminal fused to a carrier protein in living bacterial cells. The co-expression of glutamyl-charged tRNA^Glu^ in order to acylate specific Ser/Thr side chains with glutamate prior to the dehydration reaction was proposed by Zambaldo et al. (2017). The main intention was to overcome an incomplete dehydration phenomenon which occurs during incorporation of α-chloroacetamide-containing ncAA to nisin variants having altered macrocyclic topologies and antimicrobial activities. In an attempt to optimize biophysical properties of cinnamycin from *Streptococcus albus* for medical and industrial applications, three distinct pyrrolysine analogs were incorporated into two distinct positions of the antibiotic in Streptomycetes using the orthogonal pyrrolysyl-tRNA synthetase/tRNA^Pyl^ pair from *Methanosarcina barkeri*. In spite of a low rate of incorporation, the data revealed that the type of ncAA and the position of incorporation are important to achieve suitable amounts of the new deoxycinnamycin derivatives (Lopatniuk et al., 2017). Last but not least, the stereochemical configuration of thioether bridges is an important issue, since recent studies indicate that it is the property of the sequence of the core peptide and not the modification enzyme that determines the stereochemical outcome of ring formation (Garg et al., 2016).

Although most of the reports regarding the site-specific incorporation of ncAAs in RiPPs have shown lower levels of antimicrobial activity of variants than the wild type, some rare reports presented slightly improved activity on analogs (Table 2). Notably, novel modified lantibiotic variants have encrypted potential to show appropriate activity against newly emerged pathogens.

## Conclusion and Outlook

It has been estimated that 1.2 trillion USD are required to cover additional health expenditure per year expected by 2050 due to the rise of various AMRs (World Health Organization, 2020). The World Health Organization (WHO) has announced a global AMR response to coordinate this in collaboration with international partners. According to the WHO report on antimicrobial agents in clinical development stages in 2019, the majority of antibacterial agents are direct-acting small molecules (*n* = 108, 42.9%), followed by non-traditional approaches (*n* = 90, 35.7%), then AMPs (*n* = 27, 10.7%) as the main antibiotic agents (World Health Organization, 2019). It was also argued that the alarming rise in antibiotic resistance rates in the late 20th century is a clear indication that the golden age of antibiotics might be over (Wernicki, 2013). All these figures and projections urge us to reconsider current approaches and anticipate possible future paths to alternatives. In many cases, superbugs are characterized by increased multi-drug resistance, improved transmissibility and virulence, resulting in increased morbidity and mortality.

Multi-drug resistance in bacteria is a very complex problem, deeply rooted in the genetic and biochemical flexibility of bacteria, i.e., a highly pleiotropic phenomenon. Obviously, this problem cannot be solved only by simple solutions such as chemical modifications or variations of known lead substances. Therefore, we believe that the time is coming to use more extensively the most advanced methods of Synthetic Biology such as expanded genetic code to combat superbugs that have continuously evolved in most industrialized nations over the last 60 years. Here we attempted to summarize the efforts that have been made by the help of expanded genetic code to introduce new-to-nature lanthipeptides as promising alternatives to classical chemical agents. We have also tried to present new insights and progress in the field as well as the limitations and challenges which should be overcome in the future. We are firmly convinced that “radical” methods of genetic engineering (Davies and Church, 2019), such as the expansion of the genetic code, can be a useful addition to the struggle against superbugs as a global threat to all of mankind.

In the present medical context and in the current environment of failing antibiotic protection, an obvious functional expansion is a fortification against post-surgery antibiotic infections, for example by coupling therapeutic proteins to antimicrobial modules. New approaches such as genetically encoded chemical conversion (GECCO) has also been recently introduced (Yang et al., 2019) to overcome limitations of translational machinery. This and other newly emerged approaches such as OTS optimization (Dulic et al., 2018), cell-free protein synthesis (Jin and Hong, 2018), genomically recorded strain development (Amiram et al., 2015), artificial codon box division (Iwane et al., 2016), and quadruplet codon and orthogonal ribosome (d’Aquino et al., 2018) are expected to open new avenues for exploiting chemistry in live systems to bioengineering of lanthipeptides. GCE as a research field has now reached the maturity to be efficiently implemented in the bioengineering of lanthipeptides to understand their structural complexity and the behavior of the entire biosynthesis machinery. This should provide a solid basis for expanding the chemical space of recombinant AMPs. The aim is not only improving their therapeutic properties to combat AMR but also to repurpose them functionally for, e.g., anticancer or antiviral activities. The application of co-translational incorporation of ncAAs by more than 200 different chemical entities available to us for rational manipulation of various scaffolds offers unprecedented opportunities to manage the supply of advanced peptide-based antimicrobials and other sophisticated drugs in the future.

## Author Contributions

HRK-H and NB planned and conceived. All authors read, critically revised, and approved the final manuscript.

## Conflict of Interest

The authors declare that the research was conducted in the absence of any commercial or financial relationships that could be construed as a potential conflict of interest.
